# Subcellular localised small molecule fluorescent probes to image mobile Zn^2+^

**DOI:** 10.1039/d0sc04568c

**Published:** 2020-10-09

**Authors:** Le Fang, Michael Watkinson

**Affiliations:** The Joseph Priestley Building, School of Biological and Chemical Science, Queen Mary University of London Mile End Road London E1 4NS UK; The Lennard-Jones Laboratories, School of Chemical and Physical Science, Keele University ST5 5BG UK m.watkinson@keele.ac.uk

## Abstract

Zn^2+^, as the second most abundant d-block metal in the human body, plays an important role in a wide range of biological processes, and the dysfunction of its homeostasis is related to many diseases, including Type 2 diabetes, Alzheimer's disease and prostate and breast cancers. Small molecule fluorescent probes, as effective tools for real-time imaging, have been widely used to study Zn^2+^ related processes. However, the failure to control their localisation in cells has limited their utility somewhat, as they are generally incapable of studying individual processes in a specific cellular location. This perspective presents an overview of the recent developments in specific organelle localised small molecule fluorescent Zn^2+^ probes and their application in biological milieu, which could help to extend our understanding of the mechanisms that cells use to respond to dysfunction of zinc homeostasis and its roles in disease initiation and development.

## Abstract 2

A number of recently developed subcellular localised small molecule fluorescent probes to image mobile Zn^2+^ are reviewed in this perspective.

## Graphical abstract

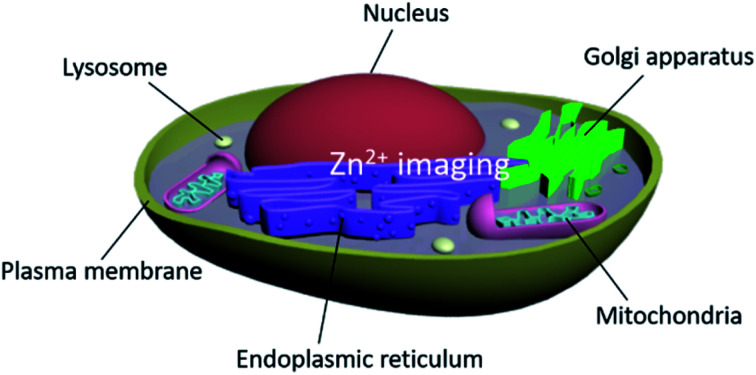

## Introduction

**Fig. 1:**
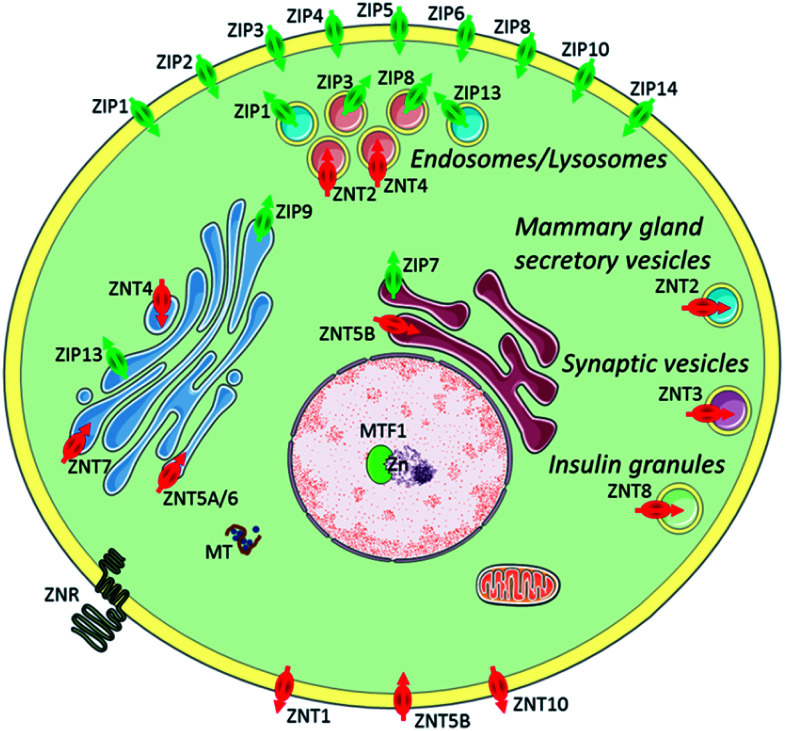
Zinc transporters (ZIP importers and ZNT exporters) and metallothioneins (MTs) to control cellular and subcellular zinc homeostasis. Reproduced from ref. 8 with permission from the Royal Society of Chemistry, Copyright [2014].

Zn^2+^ is the second most abundant d-block metal ion in the human body, with a total content of about 2 g, most of which is bound to proteins. It is estimated to exist in over three thousand proteins and is widely used for catalytic, regulatory, and structural roles.^1,2^ Zinc plays an important part in a wide range of biological processes, such as brain function and pathology, immune function, gene transcription, and mammalian reproduction. Due to this, it is unsurprising that problems with zinc homeostasis are associated with many diseases, including Alzheimer's disease,^3^ prostate cancer,^4^ type 2 diabetes,^5^ and immune dysfunction and infection.^6^ Whilst the majority of zinc is found in bound forms, there exists a pool of ‘mobile’ or ‘free’ zinc, which initiates transient signals that stimulate various physiological processes, though its concentration in the cytosol is only in the picomolar range.^7^ The thiol-rich metallothioneins (MTs) and zinc transporters are normally involved in the processes to maintain cellular zinc homeostasis.^7^ MTs acting as Zn^2+^ buffers, can bind a large amount of Zn^2+^ and release it under conditions of oxidative stress due to the antioxidant role it plays. There are two main families of zinc transporters, ZIP and ZNT, which control the import and export of cytosolic zinc to intracellular organelles or extracellular space (Fig. 1).^8^ Mobile Zn^2+^ is associated with the regulation of gene expression, insulin secretion, and is also considered as a signalling ion for intra- and intercellular communication, such as neurotransmission.^9^ Therefore, effective methods are required to image mobile Zn^2+^ at the cellular or subcellular level in order to understand these processes.^10^

**Scheme 1:**
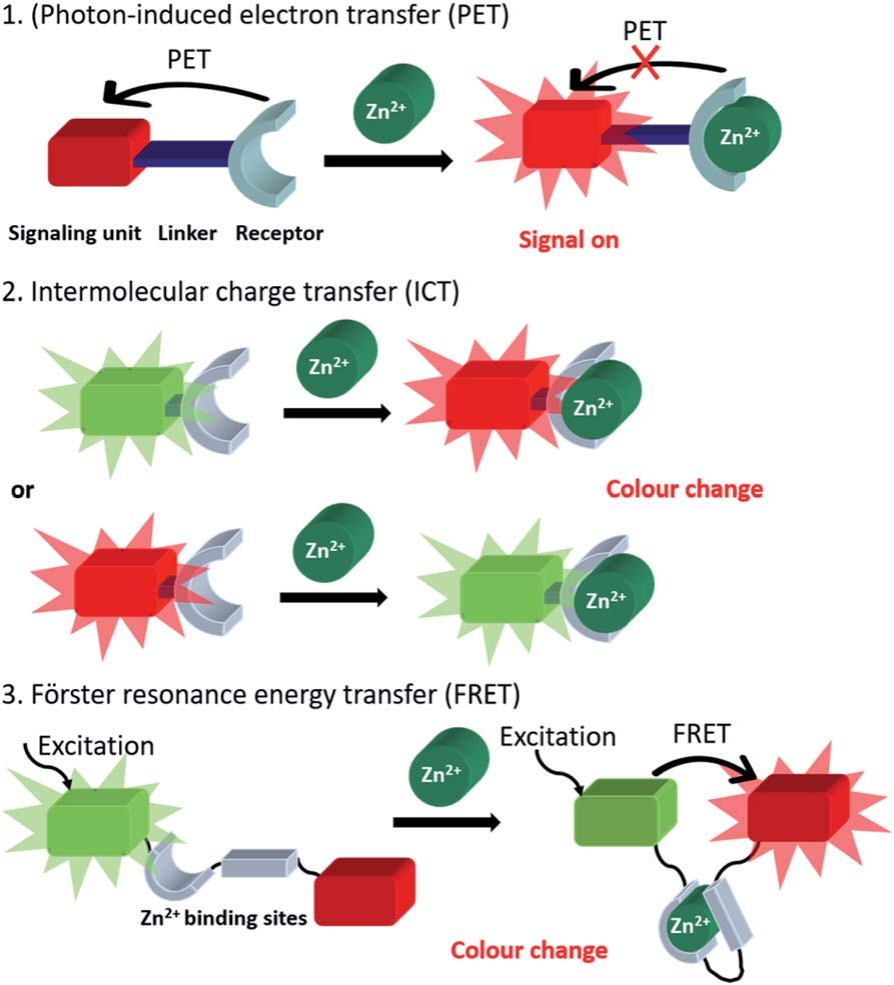
Generic representation of the three main probe classes utilised in the fluorescent imaging of mobile Zn^2+^.

Since Zn^2+^ has a d^10^ configuration, it is redox inert in biology and is rendered spectroscopically silent for most of the commonly used photo-spectroscopic techniques.^11^ As a result, the development of fluorescent chemosensors has become popular for non-invasive real-time Zn^2+^ imaging in which a change in the fluorescence intensity or wavelength occurs due to analyte binding, which leads to signal output. The most commonly used probes utilise a switch on fluorescent mechanism in which photon-induced electron transfer (PET)^12–14^ is prevented upon zinc binding (Scheme 1). Typically, such fluorescent chemosensors contain a fluorophore (the signal source), a short spacer unit and a receptor (the recognition site). In addition, fluorescent probes utilising intermolecular charge transfer (ICT)^15,16^ and Förster Resonance Energy Transfer (FRET)^17,18^ mechanisms have also been widely applied in biology. In the biological environment, fluorescent chemosensors are generally involved in a competitive exchange equilibrium with endogenous ligands and proteins and thus detect changes of mobile Zn^2+^ pools rather than total cellular zinc levels. Therefore probes displaying high sensitivity with low detection limit, high specificity and selectivity to distinguish Zn^2+^ from competing metal ions are preferred.

There are three main types of fluorescent sensors for biological zinc imaging: small molecule probes, protein-based biosensors and peptide targeting fluorescent sensors. Protein-based biosensors and peptide targeting fluorescent sensors can localise to specific organelles by introducing genetically encoded site-directing proteins and selecting specific amino acid sequences, which have high affinity for the particular targets.^10^ However, they are not without some limitations. The genetically encoded protein-based sensors cannot be transfected into all cell lines, have a small range of excitation and emission wavelengths available, as well as low photochemical stability and brightness. The peptide-based sensors are sensitive to proteases *in vivo* and cell internalization can be difficult, except for specific peptide sequences. In contrast, small molecule fluorescent probes can display high sensitivity and selectivity, low toxicity, and good photophysical properties. However, the failure to control the small molecule probes' cellular or subcellular location can limit their utility somewhat.

In the last two decades, there have been considerable efforts to overcome this problem. At the subcellular level, organelles require zinc for their normal function and the dysfunction of zinc homeostasis results in pathological processes, such as cellular stress, and these can induce cell apoptosis. Therefore, the development of Zn^2+^ probes capable of predictable and reliable subcellular localisation is required for biological applications. Reviews exist that summarize a range of subcellularly localised fluorescent probes, for cations, anions, or small molecules,^19–22^ however, a summary of the recent developments in subcellularly localised Zn^2+^ probes has, to our knowledge, not yet appeared. In this perspective, an overview of the recent development in specific organelle localised small molecule fluorescent Zn^2+^ probes is presented together with their applications in biological systems.

## Subcellular localised small molecule fluorescent Zn^2+^ probes

### Plasma membrane targeting

The plasma membrane, also known as the cell membrane, is mainly formed by a lipid bilayer to protect cells from their external environment. The membrane controls the import and export of substances and the selective uptake of ions and organic molecules. Membrane transporters, a class of membrane protein, play an important role in these processes. As shown in Fig. 1, ZIP 1–6, ZIP8, ZIP10 and ZIP14 are zinc transporters that control cellular zinc uptake, while ZNT1, ZNT5B and ZNT10 are responsible for its efflux through the membrane. Some data have suggested that when zinc is present in sufficiently high concentration it can act as a stabilizer of the cell membrane.^23^ It has also been reported that zinc deficiency in membranes causes a defect in calcium channels, which also impairs the uptake of Ca^2+^.^24^

**Fig. 2:**
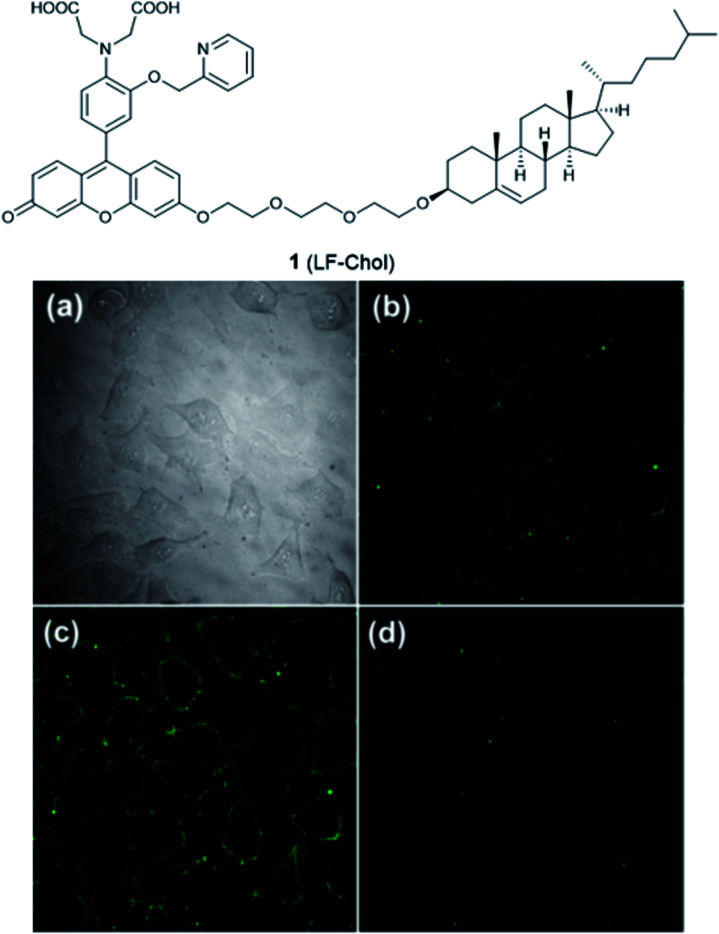
The structure of **1** (LF-Chol). (a) Bright-field transmission image of HeLa cells; (b) the fluorescence of **1** in HeLa cells; (c) the fluorescence response in the presence of 20 μM Zn^2+^ and (d) the fluorescence response after the subsequent addition of 100 μM EDTA. Reproduced from ref. 25 with permission from the American Chemical Society, Copyright [2011].

**Fig. 3:**
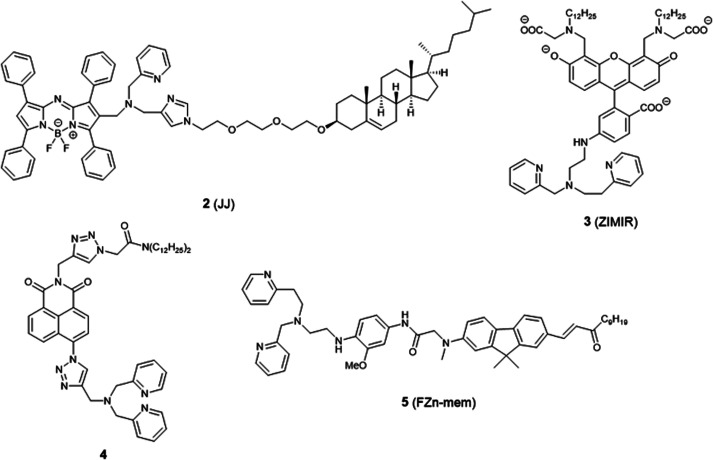
The structures of membrane localised fluorescent probes **2–5**.

There are relatively few small molecule fluorescent probes reported to image Zn^2+^ in the cell membrane. Due to the phospholipid bilayer nature of the membrane, a highly hydrophobic group is normally used as a membrane targeting unit. In 2011 Yamamoto *et al.* reported that cholesterol could be applied as a cell membrane targeting unit.^25^ With fluorescein as the fluorophore and an *o*-aminophenol-*N*,*N*,*O*-triacetic acid-based zinc-chelating moiety, probe **1** (LF-Chol) showed good cell membrane localisation and fluorescence response when Zn^2+^ was added or removed (Fig. 2). More recently, cholesterol was also applied by You *et al.* in a deep-red fluorescent probe **2** (JJ, Fig. 3) to image Zn^2+^.^26^ Probe **2** displayed good photophysical properties and tight Zn^2+^ binding with a low dissociation constant (4 pM). Co-localisation experiments in HeLa cells revealed that **2** was rapidly internalised to intracellular spaces, including the lysosome and endoplasmic reticulum (ER), but the localisation in the membrane was very low (the Pearson's coefficient was reported to be 0.12). Meanwhile, the authors found that probe **2** facilitated the influx of exogenous Zn^2+^ in the absence of the pyrithione ionophore, while a control probe without cholesterol could not, thus suggesting that the membrane targeting cholesterol unit permeabilised the cell membrane (Table 1).

**Table 1:** The photophysical properties of probes **1–34**

Probe	Solvent	Max *λ*_ex_/nm		Max *λ*_em_/nm		*ε* a/M^−1^ cm^−1^		*Φ* b		*K*_d_ c	LOD^d^
		No Zn^2+^	With Zn^2+^	No Zn^2+^	With Zn^2+^	No Zn^2+^	With Zn^2+^	No Zn^2+^	With Zn^2+^		
**1**	HEPES buffer	470	470	517	517	NR^e^	NR	<0.005	0.18	126 nM	NR
**2**	PIPES buffer	643	637	663	662	3.68 × 10^4^	3.62 × 10^4^	0.038	0.21	4 pM	NR
**3**	HEPES buffer	493	493	515	515	7.3 × 10^4^	NR	0.0032	0.225	0.45 μM	NR
**4**	HEPES buffer	347	347	420	443	NR	NR	NR	NR	NR	NR
**5**	MOPS/DPPC (1 : 1)	400	415	506	520	1.64 × 10^4^	NR	0.19	NR	20 ± 0.4 nM	NR
**6**	MOPS buffer	545	NR	575	575	NR	NR	NR	NR	65 ± 10 nM	NR
**7**	PIPES buffer	497	490	522	517	3.3 × 10^4^	4.2 × 10^4^	0.36	0.80	1.5 ± 0.2 μM	NR
**8**	PIPES buffer	569	573	629	631	2.84× 10^4^	3.67 × 10^4^	0.0355	0.5025	3.25 ± 0.12 nM	NR
**9**	MOPS buffer	388	375	500	493	1.87 × 10^4^	2.31 × 10^4^	0.15	0.92	3.1 nM	NR
**10**	HEPES buffer	413	395	536	536	1.85 × 10^4^	2.10 × 10^4^	0.0048	0.33	1.4 nM	NR
**11**	MOPS buffer	392	NR	559	NR	1.32 × 10^4^	NR	0.023	NR	17 ± 2 nM	NR
**12**	HEPES buffer	453	391	625^f^	542^f^	6.2 × 10^3^	4.2 × 10^3^	NR	0.03	8.2 ± 0.2 nM	NR
**13**	HEPES buffer	420	370	550	504	NR	NR	0.11	0.22	0.45 ± 0.01 nM	NR
**14**	CH_3_CN	347^g^	363^g^	633	633	81 × 10^3^^g^	67 × 10^3^^g^	0.28^i^	0.29^j^	9.1 μM	NR
		602^h^	602^h^			23 × 10^3^^h^	21 × 10^3^^h^				
**15**	PIPES buffer	510	510	535	529	NR	NR	<0.001	0.75 ± 0.03	0.6 ± 0.03 nM	NR
**16**	HEPES buffer	496	469	550	534	2.10 × 10^4^	1.40 × 10^4^	0.003	0.046	1.0 ± 0.1 nM	NR
**17** k	MES buffer	285	261	542	495	4.30 × 10^3^	1.66 × 10^3^	0.11	0.17	16 ± 1.1 nM	NR
**18** l	MES buffer	435	444	528	536	1.55 × 10^4^	1.81 × 10^4^	0.03	0.23	8.5 μM	0.18 μM
**19**	EtOH/Tris–HCl (9 : 1)	597	568	647	578	NR	NR	NR	0.23	68 ± 4 μM^m^	0.48 μM
										123 ± 6 μM^n^	
**20**	MES buffer^o^	500	490	536	536	NR	NR	NR	NR	NR	0.477 μM
**21a**	HEPES buffer/CH_3_CN (1 : 1)	350	365	504	504	NR	NR	0.25	0.24	1 nM^2^	NR
**21b**		350	359	504	504	NR	NR	0.32	0.33	0.17 nM^2^	NR
**22a**	HEPES buffer	456	454	515	515	NR	NR	0.0027	0.0482	1.54 nM	NR
**22b**	HEPES buffer	454	451	509	515	NR	NR	0.0064	0.2606	2.57 nM	NR
**22c**	HEPES buffer	453	453	512	517	NR	NR	0.0077	0.3283	1.91 nM	NR
**23a**	PIPES buffer	478	530	625	628	1.93 × 10^4^	2.64 × 10^4^	0.067	0.41	0.69 nM	NR
**23b**	PIPES buffer	480	524	630	630	1.69 × 10^4^	2.56 × 10^4^	0.069	0.22	0.70 nM	NR
**23c**	PIPES buffer	480	535	623	628	1.33 × 10^4^	1.93 × 10^4^	0.342	0.60	<0.001 nM	NR
**24**	EtOH	406	NR	490	NR	NR	NR	<0.01	0.12	5.5 nM	5.8 nM
**25**	HEPES buffer	346	346	414	414	NR	NR	0.041	0.25	3.5 nM	47 pM
**26**	HEPES buffer	346	346	414	414	NR	NR	0.013	0.041	4.7 nM	0.71 nM
**27**	HEPES buffer	346	346	414	414	NR	NR	0.05	0.28	2.83 ± 0.11 nM	48 pM
**28**	PIPES buffer	515	507	530	525	7.95 × 10^4^	8.40 × 10^4^	0.39	0.87	0.7 ± 0.1 nM	NR
**29a**	PIPES buffer	499	496	519	514	2.72 × 10^4^	1.60 × 10^4^	0.004	0.73	p	NR
**29b**	PIPES buffer	498	492	515	515	6.41 × 10^4^	4.00 × 10^4^	0.012	0.51	q	NR
**30**	HEPES buffer	302	360	532	532	420	4680	0.015	0.055	1.8 ± 0.1 pM	0.042 pM
**32a**	MOPS buffer	384	388	518	518	2.89 × 10^4^	NR	0.12^l^	0.93^l^	1.7 nM^r^	NR
**33**	HEPES buffer	453	442	546	536	NR	NR	NR	0.19	4 nM	57 nM
**34**	HEPES buffer	346	346	414	414	NR	NR	0.02	0.05	18.8 nM	NR

a Molar extinction coefficient.

b Fluorescence quantum yield.

c Dissociation constant.

d The limit of detection.

e Not reported.

f *λ*_ex_ = 466 nm.

g The parameters of donor.

h The parameters of acceptor.

i *λ*_ex_ = 565 nm.

j *λ*_ex_ = 400 nm.

k All results shown here were recorded at lysosomal pH 5.2.

l All results shown here were recorded at lysosomal pH 5.0.

m Calculated from turn on fluorescence emission at wavelength 578 nm.

n Calculated from the ratio of *F*_578 nm_/*F*_680 nm_.

o The pH of the buffer is 5.0.

p *K*_d1_ = 3.5 ± 0.1 mM, *K*_d2_ = 150 ± 100 μM.

q *K*_d1_ = 220 ± 30 μM, *K*_d2_ = 160 ± 80 μM, *K*_d3_ = 9 ± 6 μM.

r The results were obtained in solution EtOH : MOPS buffer (1 : 1, v/v).

Besides cholesterol, long alkyl chains have also been widely used for membrane targeting. Rutter, Li *et al.* reported the fluorescent probe **3** (ZIMIR) in which a pair of dodecyl alkyl chains were used as the membrane targeting unit as a Zn^2+^ indicator to image dynamic insulin release.^27^ The probe was quenched based on the PET mechanism and displayed a robust fluorescence increase after binding with Zn^2+^ and showed low toxicity and membrane labelling in a wide range of cell lines. Using this probe, the authors demonstrated exocytotic activity at subcellular resolution from pancreatic β cells in intact islets and found that the sites of Zn^2+^/insulin release are mainly in small groups of adjacent β cells. Similar results were observed by Watkinson *et al.* with the plasma membrane targeting Zn^2+^ probe **4**.^28^ The di-dodecylamide motif as the membrane targeting unit was introduced through a one-pot modular ‘click-S_N_Ar-click’ approach, which was shown to be more efficient in the synthesis. Probe **4** showed a significant switch on fluorescence response to Zn^2+^ due to aggregation phenomena, and *in cellulo* experiments in mouse pancreatic islets demonstrated its localisation to the exterior of the plasma membrane. This probe was subsequently used to measure dynamic insulin secretion by Hodson *et al.* since zinc is co-released from insulin-containing granules.^29^

Similarly, Cho *et al.* synthesized the two-photon (TP) probe, **5** (FZn-mem), to image near-membrane Zn^2+^ by introducing a long alkyl chain to 2-amino-7-(3-oxo-1-dodecen-1′-yl)-9.9-dimethylfluorene (ADF), a two-photon fluorophore with a large TP cross section.^30^ With the same metal-binding motif as **3**, probe **5** was shown to be highly selective for Zn^2+^ over competing cations, and its dissociation constant *K*_d_ was determined to be 20 ± 0.4 nM and 19 ± 0.2 nM by one-photon and two-photon spectroscopy, respectively. After TP excitation by 820 nm femtosecond laser pulses, the fluorescence of **5** could be collected in the emission wavelength range of 450–600 nm, and near-membrane Zn^2+^ could be detected in living cells and tissue at a depth of 110 μm.

### Mitochondria targeting

The mitochondrion is an important organelle in eukaryotic organisms. It generates most of the chemical energy supply of adenosine triphosphate (ATP) in all cells, and takes part in many biological processes, such as signalling, the cell cycle, cell growth and cell death.^31^ Mitochondria are involved in intracellular Zn^2+^ storage and as a co-factor for a wide range of enzymatic reactions and Zn^2+^ is closely related to the mitochondrial respiratory chain.^32^ It was also reported that zinc can reduce mitochondrial damage under stress conditions, which can protect cells from oxidatively-induced apoptosis.^33^ In contrast, mitochondrial dysfunction may cause rapid Zn^2+^ entry, which is one of the major contributors to neuronal injury.^34^ Therefore, to understand these processes better, mitochondria localised zinc imaging is in demand.

**Fig. 4:**
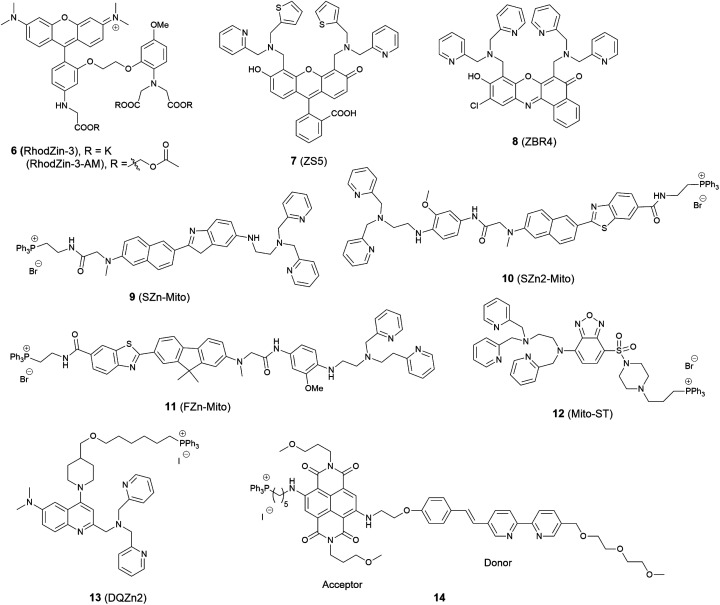
The structures of mitochondria localised fluorescent probes **6–14**.

The first example of a mitochondrial fluorescent Zn^2+^ probe was reported by Gee *et al.* in 2003.^35^ Probe **6** (RhodZin-3, Fig. 4) displayed a 75-fold fluorescence increase after binding Zn^2+^ and good selectivity over competing cations. The ester form was loaded into cells due to its membrane permeability and it showed co-localisation with a mitochondrial marker MitoTracker Green and Zn^2+^ response in cortical neurons.

In 2006, Lippard *et al.* synthesized a series of probes and found **7** (ZS5), displaying green fluorescence, could localise to mitochondria^36^ although this was the only probe to display mitochondrial localisation within a larger series of structurally similar probes synthesized. Another probe **8** (ZBR4), a red fluorescent probe, was subsequently found to spontaneously accumulate in mitochondria in 2014 by the same group.^37^ However, the localisation was again apparently serendipitous as this was the only probe in another large family of probes that failed to localise to the endoplasmic reticulum (ER).

**Fig. 5:**
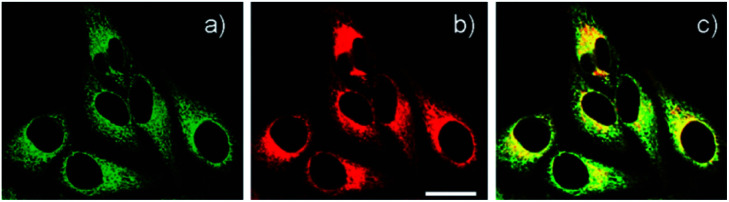
(a) TPM and (b) OPM images of HeLa cells co-labelled with (a) **9** (0.5 μM) and (b) Mitotracker Red FM (1 μM) for 30 min; (c) colocalised image. Reproduced from ref. 38 with permission from the American Chemical Society, Copyright [2011].

Delocalised lipophilic cations, such as the triphenylphosphonium salt (TPP), are known as effective mitochondrial targeting groups, since they can cross hydrophobic membranes and accumulate in the mitochondria in living cells. By introducing this group Kim and Cho *et al.* obtained a mitochondria-targeting two-photon probe **9** (SZn-Mito) for Zn^2+^ imaging (Fig. 5).^38^ The probe showed a 7-fold increase of two-photon excited fluorescence after being bound with Zn^2+^ and was able to detect zinc in living tissues to a depth of 100–200 μm through two-photon microscopy. Just one year later, the same group developed a similar mitochondria targeting probe **10** (SZn2-Mito) which had a 70-fold two-photon excited fluorescence increase in response to Zn^2+^ and displayed similar properties.^39^ Besides these, another two-photon Zn^2+^ probe **11** (FZn-Mito) was synthesized with a different fluorophore, but it had similar mitochondria targeting behaviour and two-photon emission response to Zn^2+^.^40^

In 2012 Guo *et al.* synthesized the ratiometric probe **12** (Mito-ST) which was capable of imaging zinc ions in the mitochondria.^41^ With Zn^2+^ associated, the probe showed a blue shift in both excitation and emission wavelength. Interestingly, they observed the concentration of Zn^2+^ in mitochondria started to increase immediately upon H_2_O_2_ (10 mM) stimulation and increased to 1.6 nM (from 0.6 nM) with the ratiometric imaging result based on the method reported by Tsien *et al.*^42^ However, the Zn^2+^ release stimulated by *S*-nitrosocysteine (SNOC, 10 mM), a precursor of the NO radical, experienced a lag phase, and the concentration was much higher (76 nM) compared to that stimulated by H_2_O_2_. In the same year Jiang *et al.* reported the quinoline-based ratiometric probe **13** (DQZn2) with similar properties.^43^ The emission of the probe showed a 46 nm blueshift, with about a 5-fold change of the emission intensity ratio after binding with Zn^2+^, which allowed the concentration of mitochondrial free Zn^2+^ to be quantitatively measured in NIH3T3 cells.

The mitochondria localised Zn^2+^ probe **14** utilising a FRET mechanism was reported by Zhu *et al.*^44^ The FRET donor fluorophore displayed a bathochromic shift of emission when coordinated with Zn^2+^. The spectral overlap between the emission of the donor and the absorption of the acceptor increased upon Zn^2+^ binding, which enhanced the FRET efficiency. In HeLa cells, when the probe was excited at 405 nm, red fluorescence with emission wavelength 580–680 nm could be observed after addition of ZnCl_2_.

**Fig. 6:**
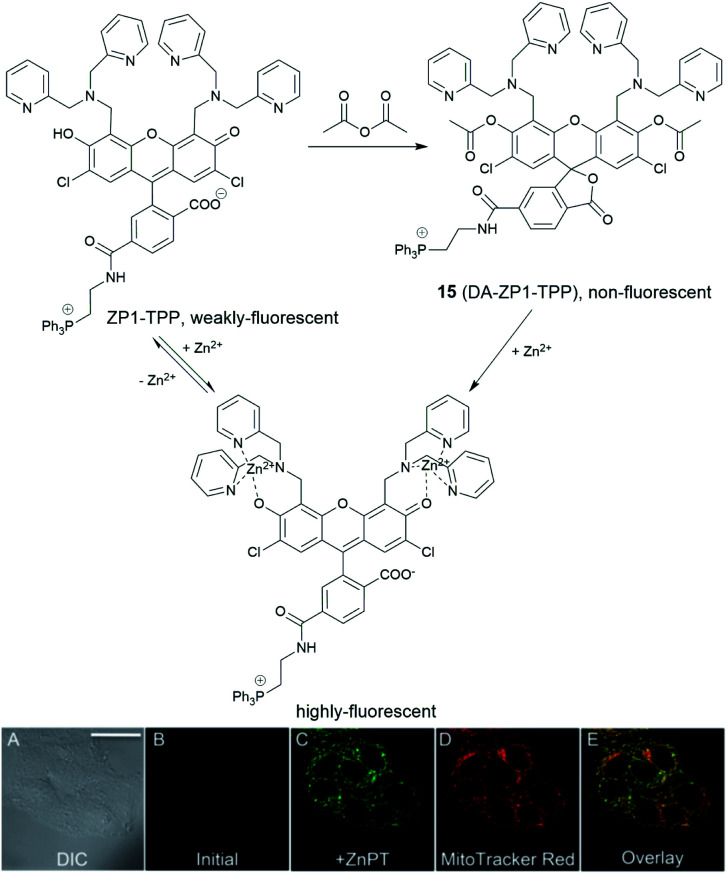
The structure of the reaction-based probe **15** (DA-ZP1-TPP). Its fluorescence response to Zn^2+^ and co-localisation with Mito-tracker red. Reproduced from ref. 45 with permission from the National Academy of Sciences, Copyright [2014].

In 2014, a reaction-based fluorescent probe **15** (DA-ZP1-TPP) was reported to investigate mobile Zn^2+^ in mitochondria.^45^ As shown in Fig. 6, the phenolic oxygen atoms of the xanthene ring in the fluorescein moiety was protected with an acetyl group, which rendered it non-fluorescent in metal-free media. In the presence of Zn^2+^, the ester group was hydrolysed, and the PET mechanism quenched by Zn^2+^ association, giving a strong fluorescence response (more than a 140-fold increase). Interestingly, using this probe, the authors found that tumorigenic epithelial prostate cells could not accumulate mobile Zn^2+^ in their mitochondria, compared to that of healthy epithelial prostate cells, which could.

### Lysosome targeting

The lysosome acts as the waste disposal system in cells by digesting unwanted materials in the cytoplasm. Zn^2+^ homeostasis in the lysosome is regulated by zinc transporters, ZIP3, ZIP8 and ZNT2, and its dysregulation is associated with a wide range of pathological processes at the cellular level. For example, oxidative stress induced by H_2_O_2_ causes Zn^2+^ accumulation in the lysosome in hippocampal neurons, which eventually undergo lysosomal membrane permeabilization (LMP) and this may be the mechanism of oxidative neuronal death.^46^ It was also reported that lysosome-related organelles in intestinal cells of *C. elegans* (gut granules) are the major site of zinc storage, which promotes detoxification and subsequent mobilization, regulating cellular and organismal zinc metabolism.^47^ Therefore, it is vital to develop fluorescent probes to image Zn^2+^ in the lysosomal space to help understand these kinds of biological processes.

**Fig. 7:**
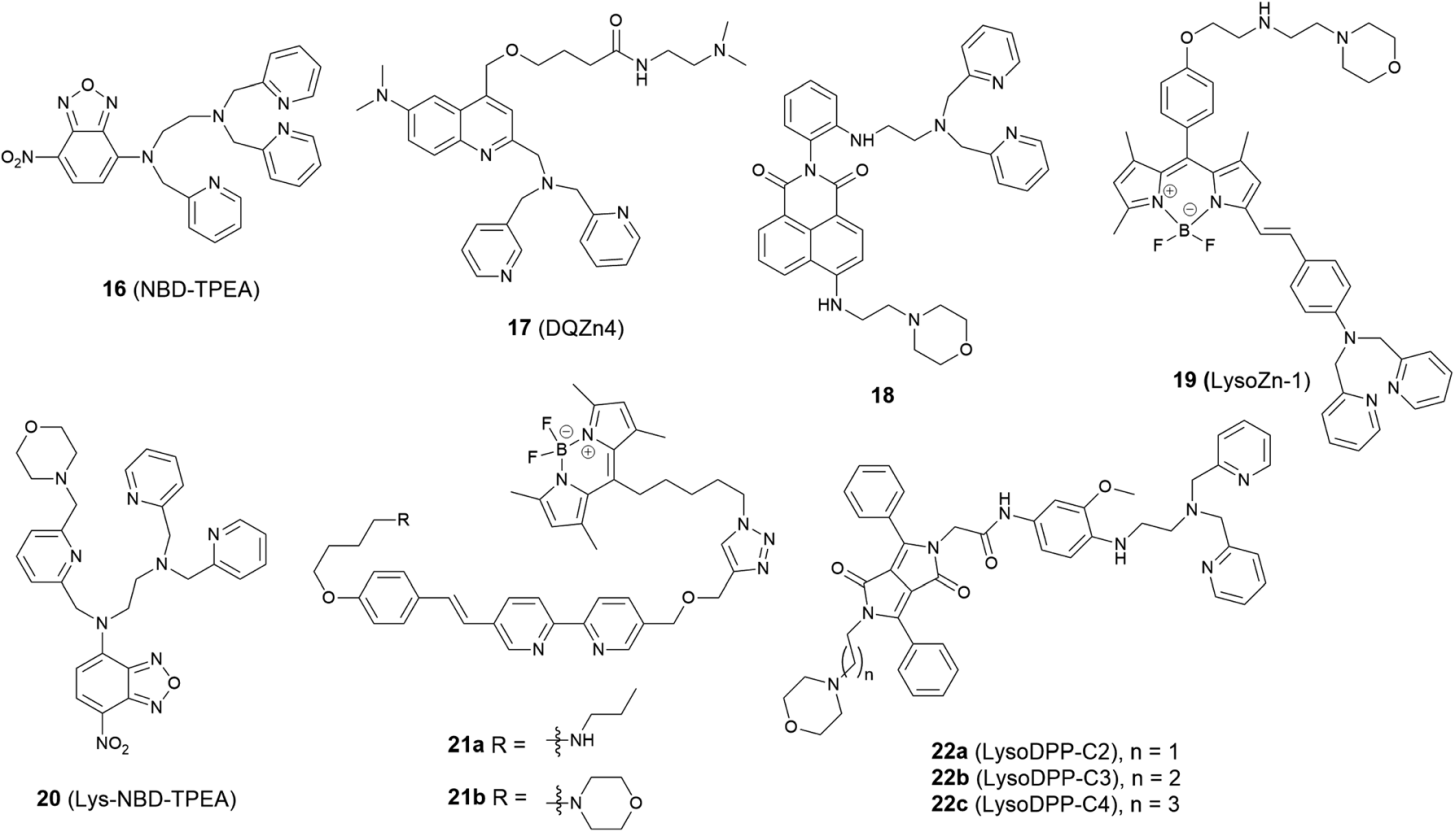
The structures of lysosome localised fluorescent probes **16–22**.

In 2009 Guo *et al.* synthesized the intramolecular charge transfer (ICT) based probe **16** (NBD-TPEA, Fig. 7).^48^ The probe demonstrated a good selectivity for Zn^2+^, a large Stokes shift, and was also applied for *in vivo* Zn^2+^ imaging in zebrafish larva. However, subcellular experiments showed it to not only accumulate in the lysosome but also in the Golgi apparatus, which may perhaps limit its application in lysosomal Zn^2+^ imaging.

The basic ethylenediamine group has been reported to accumulate in the lysosome since it can be protonated by the acid environment in lysosomal space (pH = 4.5–5.5). The basic dimethylethylamino moiety has therefore been introduced as a lysosome targeting group in the ratiometric probe **17** (DQZn4) based on quinoline and showed good targeting behaviour.^49^ It performed well in the acidic pH environment, showing a significant turn-on fluorescence response and 47 nm blueshift of emission wavelength on binding Zn^2+^. With these desirable properties *in vitro*, it was successfully utilised for imaging lysosomal Zn^2+^ changes in NIH 3T3 cells.

Another group that has been frequently used for targeting the lysosome is the morpholine unit. One example is the two-photon probe **18** based on a naphthalimide dye with an *N*,*N*-di-(2-picolyl)ethylenediamine (DPEN) Zn^2+^ ligand, which can image intracellular Zn^2+^ in the lysosome and mouse brain tissues under two-photon excited microscopy.^50^ Unlike the other subcellular targeting probes, in this case organelle differentiation relied on a concentration gradient of the probe intracellularly. Probe **18** showed a strong fluorescence switch on response to Zn^2+^ in the lysosomal pH range (pH = 4.5–5.5) but the intensity increase was much smaller at cytosolic pH range (pH = 7.2–7.4), therefore it was able to detect lysosomal Zn^2+^ specifically.

**Fig. 8:**
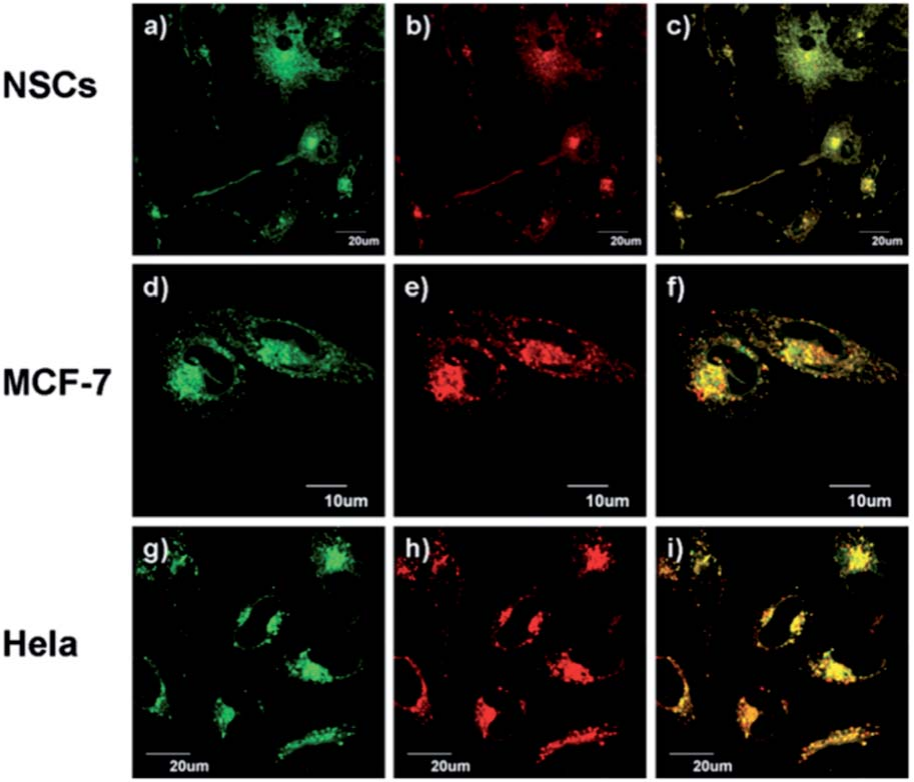
**19** (LysoZn-1) co-localises to lysosomes in NSCs (a–c), MCF-7 (d–f) and HeLa (g–i) cells. Cells were stained with LysoSensor Green (1 μM) (a, d and g), and **19** (1 μM) (b, e and h), (c, f and i) Overlap of these two channels. Reproduced from ref. 51 with permission from the Royal Society of Chemistry, Copyright [2014].

Using the same targeting unit Peng *et al.* reported a ratiometric probe **19** (LysoZn-1) to image lysosomal Zn^2+^ (Fig. 8).^51^ The authors introduced an electron donor 4-ethoxylphenyl to the *meso*-position of a styryl-Bodipy-DPA scaffold, which distinguished Zn^2+^ from Cd^2+^ very well. They explained this using Hard-Soft Acid–Base theory,^52^ which was supported by theoretical calculations. Probe **19** exhibited a significant fluorescence increase and ratiometric (*F*_578 nm_/*F*_680 nm_) changes upon Zn^2+^ binding and had a good response to Zn^2+^ in the lysosomal pH range. Using **19** it was observed that lysosomal Zn^2+^ concentration increased upon H_2_O_2_ stimulation in neuronal stem cells and a similar phenomenon was observed by Uvdal *et al.* with a PET based lysosome localised probe **20** (Lys-NBD-TPEA) in which morpholine was again utilised as the targeting unit.^53^

**Fig. 9:**
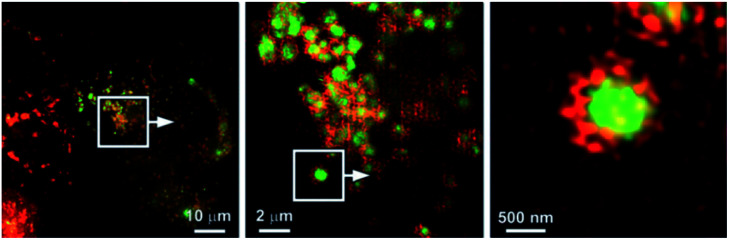
SIM images to show the localisation of compound **21b** (green) compared to that of the lysosome-associated membrane protein (LAMP) fused to the fluorescent protein FusionRed (red). Reproduced from ref. 54 with permission from Wiley-VCH, Copyright [2015].

Based on a FRET mechanism, two fluorescent Zn^2+^ probes **21** were prepared by Zhu *et al.* with different aliphatic amino groups as the lysosome targeting units.^54^ A Zn^2+^-sensitive arylvinylbipyridyl fluorophore was selected as the FRET donor, and through the efficient intramolecular FRET process, its broad emission band was transformed into a strong, narrow emission band of the acceptor Bodipy, which is preferable for multi-colour imaging. With a 2 : 1 stoichiometry between **21** and Zn^2+^, the molar absorptivity of the donor was increased upon Zn^2+^ coordination, leading to the enhancement of acceptor emission. The lysosome localisation was confirmed by confocal microscopy and with the high resolution of structured illumination microscopy (SIM), they found **21b** localised to the interior of lysosomes in HeLa cells, rather than anchoring at the lysosomal membranes (Fig. 9). Sessler *et al.* synthesized a series of probes **22** (LysoDPP-C2-C4), which were designed based on AND logic to detect both Zn^2+^ and the acidic pH of the lysosome.^55^ The morpholine moiety served not only as a lysosome targeting unit but also as a pH-responsive marker, since the nitrogen in the morpholine moiety was protonated at low pH and the PET was quenched, which increased the fluorescence of the diketopyrrolopyrrole (DPP) fluorophore in the same way as the PET quenching upon Zn^2+^ binding. The experiments *in cellulo* showed **22c** was the most effective probe in terms of the initial fluorescence and the fluorescence response to Zn^2+^. When incubated with **22c**, the cancerous prostate cell lines PC3 and DU145 showed no change in fluorescence intensity, while the normal human prostate epithelial cell line RWPE1 displayed a significant increase on the addition of exogenous Zn^2+^. The authors also demonstrated that the probe was capable of imaging the prostate *in vivo* nude mice models and discriminating between cancerous and normal prostate tissue through histological studies.

### Endoplasmic reticulum (ER) targeting

The ER, an organelle in eukaryotic cells, serves a number of important cellular roles, such as protein synthesis and transport, protein folding, carbohydrate metabolism, lipid and steroid synthesis.^56^ Proteins synthesized in the ER are normally properly folded and transported to the Golgi apparatus, however, when there are changes to ER function, resulting from factors such as ageing, genetic mutations, or the environment, unfolded or misfolded proteins are synthesized and accumulate in the ER, causing ER stress, which activates the unfolded protein response (UPR).^57^

It is known the ER acts as an intracellular store for biological mediators, including zinc, which it requires for normal function. For example, it has been found that zinc can be released from thapsigargin- and inositol 1,4,5-trisphosphate (IP3)-sensitive ER storage in cortical neurons.^58^ ZIP7, ZIP9, ZIP13 and ZNT5-7 are the transporters to regulate Zn^2+^ inside the ER and the depletion of zinc transporters and zinc deficiency can cause ER stress and upregulate the UPR,^59,60^ which can result in inflammation^61^ and a wide range of diseases, such as diabetes^62^ and neurodegenerative disorders, including Parkinson's and Alzheimer's diseases.^63^ However, the role of ‘mobile’ zinc in these processes is little understood due to the lack of suitable molecular tools to image this subcellular region that exist.

**Fig. 10:**
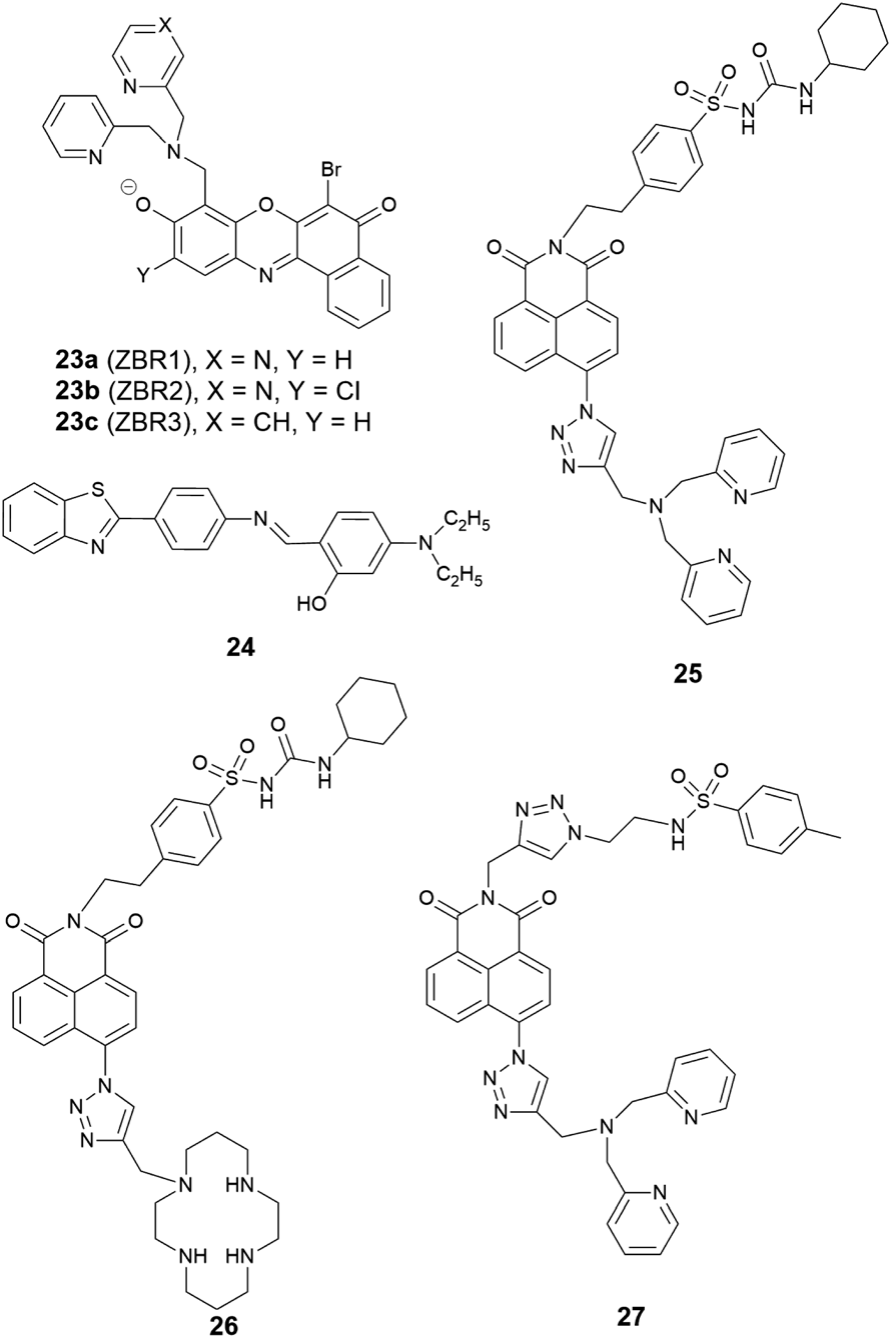
The structures of ER localised fluorescent probes **23–27**.

**Fig. 11:**
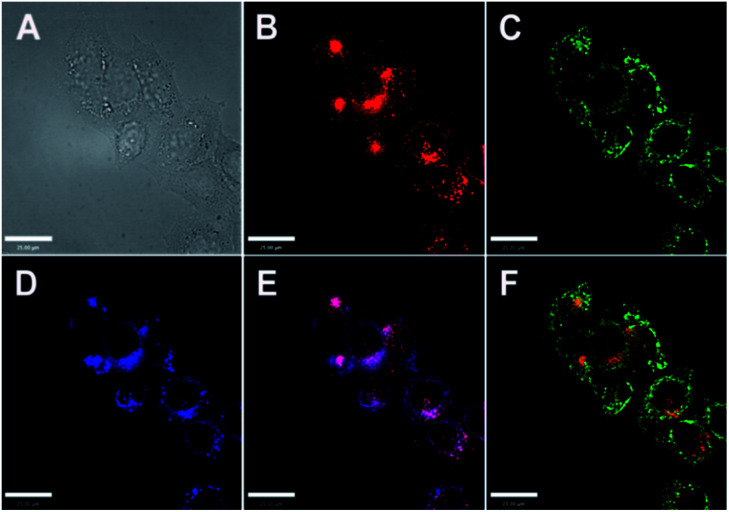
Co-localisation analysis of **23a** (ZBR1) with organelle-specific markers in HeLa cells (A) bright-field image; (B) **23a** (5 μM); (C) Mito-Tracker Green (0.2 μM); (D) ER-Tracker (1 μM); (E) overlay of **23a** and ER-Tracker; (F) overlay of **23a** and Mito-Tracker Green. Scale bar = 25 μm. Reproduced from ref. 64 with permission from the American Chemical Society, Copyright [2013].

Reports of ER localised small molecule Zn^2+^ probes are very limited, and all early reports were of systems found to accumulate in the ER adventitiously. In 2013 Lippard *et al.* reported a series of benzoresorufin based red-emitting fluorescent probes **23** (the ZBR family, Fig. 10) for labile Zn^2+^.^64^ The probes displayed a broad absorption band and a bathochromic shift after binding with Zn^2+^, while the emission showed up to 8.4-fold increase with addition of Zn^2+^. *In cellulo* studies revealed all probes accumulated in the ER in a variety of cell lines, as the Pearson's correlation coefficient with ER tracker was much higher the other organelle tracker dyes (Fig. 11). Interestingly, with **23a**, the authors observed the depletion of labile zinc in the ER of neural stem cells under ER stress induced by peroxynitrite.

The conveniently prepared small molecular fluorescent probe **24** included benzothiazole as the fluorophore and an *o*-hydroxyl Schiff base as the Zn^2+^ receptor.^65^ Upon binding Zn^2+^, the PET process was blocked, and the fluorescence response had a 65-fold increase with a slight red shift, with a Job's plot showing a 2 : 1 binding stoichiometry between **24** and Zn^2+^. In human liver hepatocellular carcinoma cells, **24** was able to detect intracellular endogenous Zn^2+^ and its subcellular localisation was found to be the ER with a Pearson's correlation coefficient of 0.88 compared to ER tacker Red. Moreover, **24** was applied to investigate Zn^2+^ in plant tissues where it was shown that Zn^2+^ mainly accumulates in vascular tissue and epidermal cells in the cotyledons when treated with exogenous Zn^2+^.

Given the ongoing need to develop an effective and reliable strategy for targeting the ER, a number of targeting units for the organelle have been explored. Glibenclamide functions as the ER targeting group in the commercial ER tracker red and ER tracker green, and using a sulfonyl urea analogue, we developed two probes **25** and **26** as ER targeting probes.^66^ It was shown that both probes accumulated in the ER in a number of cell lines, and displayed a good switch on fluorescence response to Zn^2+^. Probe **25** was used to demonstrate that a decrease of mobile Zn^2+^ concentration in the ER occurs under conditions of ER stress induced by tunicamycin and thapsigargin.

In addition to this cyclohexyl sulfonylurea moiety, the recently reported methyl phenyl sulfonamide was also incorporated as an ER targeting group through an alternative modular ‘click-S_N_Ar-click’ approach.^67^ The probe obtained, **27**, was also found to localise to the ER compared to the other organelles tested.

### Golgi apparatus targeting

The Golgi apparatus works as a central station in cells, receiving secretory cargoes exported from the ER packing proteins into membrane-bound vesicles and sending them to their intra- and extra-cellular destinations. Zn^2+^ is integral to these processes for a variety of proteins, functioning in catalytic, regulatory, and structural roles. For example, it was found that zinc takes part in the interaction between the two main Golgi proteins GRASP55 and Golgin45, maintaining the normal morphology of the Golgi apparatus;^68^ Zn^2+^ also coordinates with insulin monomers in the trans-Golgi network to package it into secretory granules, which are then released from pancreatic β-cells.^69^ To regulate Zn^2+^ homeostasis in the Golgi apparatus, transporters ZNT4-7 and ZIP7, ZIP9, ZIP11, ZIP13 are responsible for Zn^2+^ import and export, respectively. The breakdown of Zn^2+^ homeostasis in the Golgi apparatus is likely to be associated with a range of human disorders, such as cancer and neuronal, liver, kidney and eye diseases^70^ and the development of effective methods for its detection and monitoring are required.

**Fig. 12:**
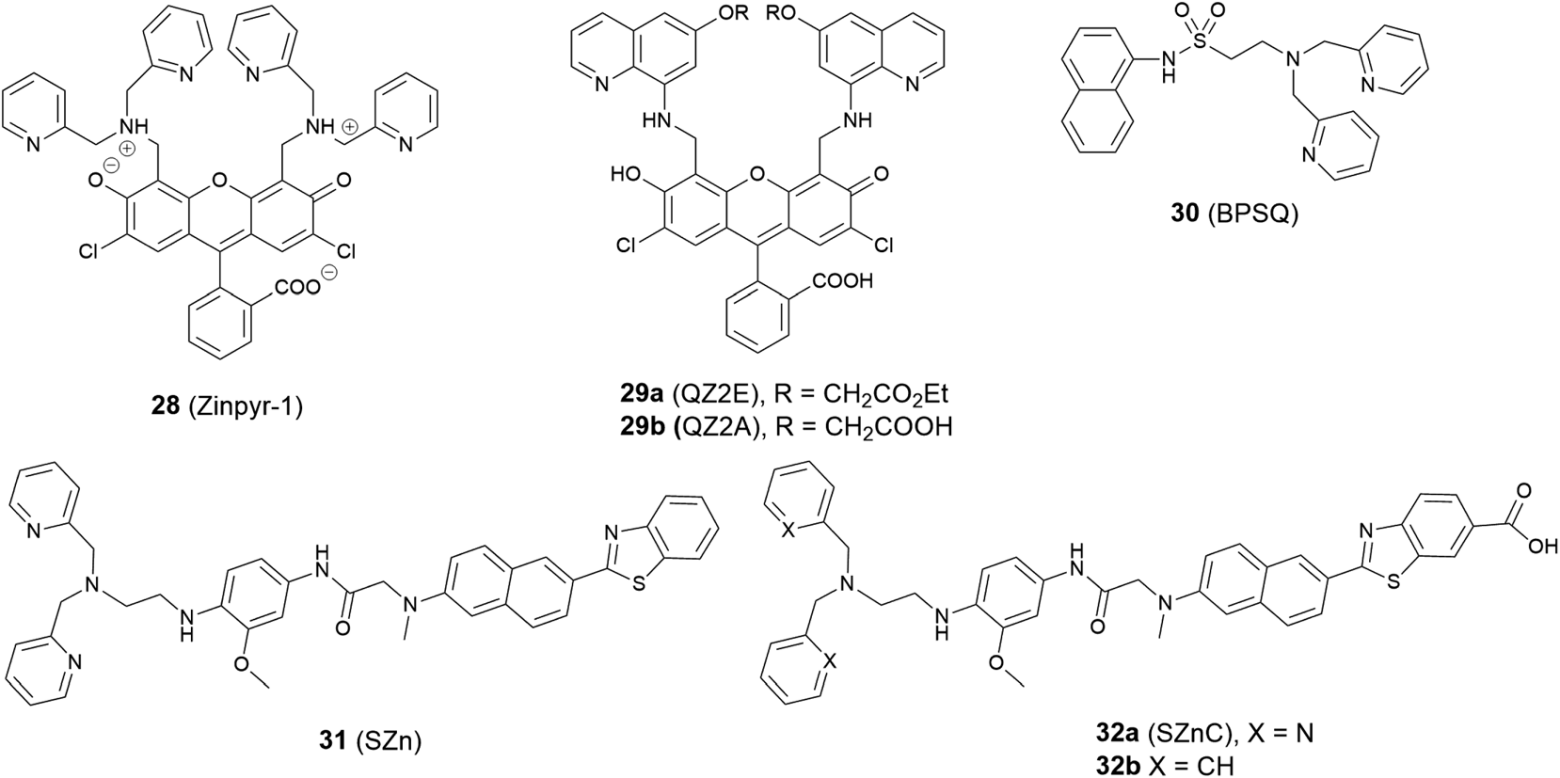
The structures of Golgi apparatus localised fluorescent probes **28–32**.

The first Zn^2+^ probe to localise in the Golgi apparatus was reported in 2000 by Lippard, Tsien *et al.*^71^ With fluorescein as the signalling unit, probe **28** (Zinpyr-1, Fig. 12) has a large extinction coefficient, high quantum yield, and good membrane permeability. Experiments in Cos-7 cells showed it colocalised well with a galactosyl transferase-enhanced cyan fluorescent protein fusion (GT-ECFP), confirming **28** stains the Golgi apparatus. Some years later Lippard *et al.* presented two cell-trappable fluorescent Zn^2+^ probes: a carboxylic ester probe **29a** (QZ2E) and its carboxylic acid analogue **29b** (QZ2A).^72^ The probes were poorly emissive but had a significant increase in emission after binding with Zn^2+^ (120-fold for **29a**, 30-fold for **29b**). Interestingly, the authors found the **29b** was cell membrane-impermeable due to its carboxylic acid moieties, but **29a** was membrane permeable and mainly localised to the Golgi apparatus; after 18 hours' incubation time, the ester was hydrolysed to produce **29b**, which was trapped inside cells.

Guo *et al.* modified the 8-sulfonamidoquinoline based commercial probe Zinquin to produce a new fluorescent probe **30** (BPSQ) to image Zn^2+^ with a 1 : 1 binding stoichiometry, which was better at discriminating mobile Zn^2+^.^73^ Co-localisation experiments showed it to accumulate preferentially in the Golgi apparatus.

**Fig. 13:**
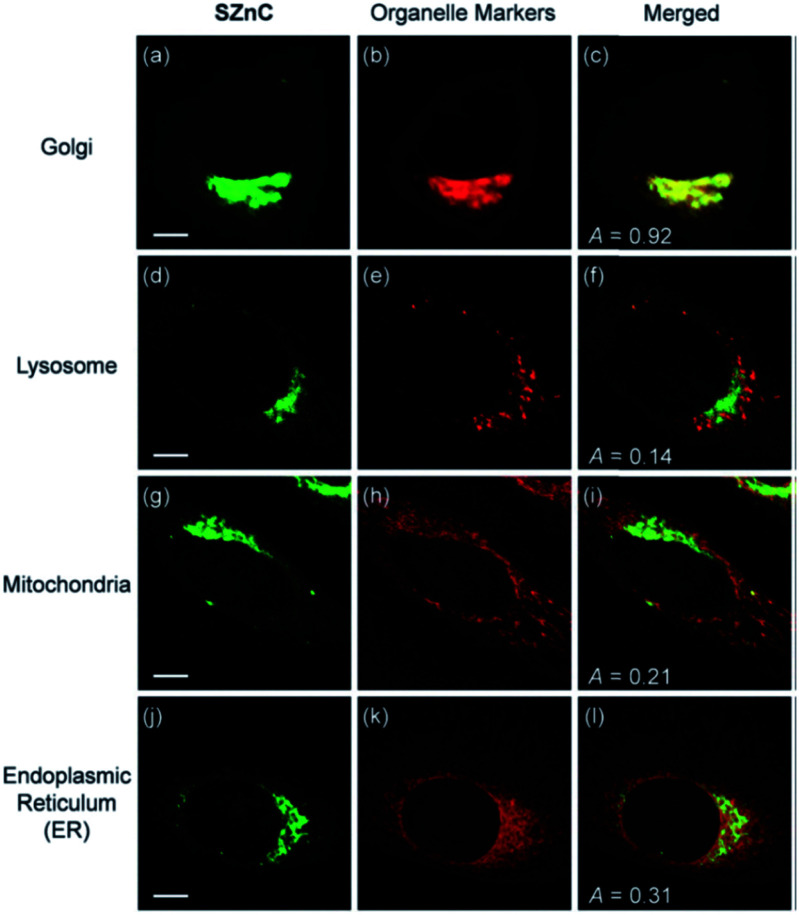
The colocalisation of probe **32a** (SZnC) (2 μM) and organelle markers in HeLa cells. Reproduced from ref. 74 with permission from Royal Society of Chemistry, Copyright [2015].

Following the previous experience on mitochondria targeting Zn^2+^ probes,^38,39^ Kim *et al.* reported a Golgi-localised two-photon probe **32a** (SZnC), which had a strong two-photon excited fluorescence enhancement in response to Zn^2+^ for real-time monitoring of Golgi Zn^2+^ changes (Fig. 13).^74^ According to theoretical predictions for Golgi apparatus staining, the lipophilicity value (log *P* value) should be within the range of 3–5.^75^ The log *P* values were calculated through measuring the probe's partitioning ratio between *n*-octanol and buffer, and the value of **32a** was found to be 2.9 ± 0.1, which is reasonably matched to the theoretical range whereas it was determined to be 2.5 ± 0.1 for the control compound **31** (SZn), which experiment proved to be non-Golgi targeting. However, the log *P* values of another control probe **32b** was 3.1 ± 0.1 and this was spread over the entire cell except the nucleus. The authors attributed the Golgi localisation of **32a** to the weakly basic pyridyl group, which **32b** does not possess. Therefore, both the lipophilicity and pyridyl moiety of **32a** appear to contribute to its accumulation in the Golgi apparatus and the probe was applied for imaging Zn^2+^ in rat hippocampal tissue and could be observed at a depth of more than 100 μm with two-photon microscopy. In addition to the log *P* value, it may be that other factors, such as amphiphilicity, electric charge and p*K*_a_ values, molecular and ionic weights and conjugated bond number of the probes could also be considered to predict their location more accurately.^75^

It appears that all of the probes described above were found to reside in the Golgi apparatus adventitiously, therefore a Golgi targeting unit is still required in order to develop reliable Golgi apparatus localised Zn^2+^ probes. Ceramide may be an option for Golgi apparatus targeting since it has been employed in commercial stains for the Golgi apparatus such as NBD-ceramide and Bodipy-ceramide. However, it requires multi-step synthesis and also co-localises to the plasma membrane, which may limit future applications to some extent. More recently a phenylsulfonamide-based fluorescent probe was reported to localise to the Golgi apparatus for H_2_S imaging,^76^ which may provide a possible Golgi apparatus targeting unit for Zn^2+^ probes. Furthermore, cysteine has been reported as a Golgi apparatus targeting unit in other systems,^77,78^ since galactosyltransferase and protein kinase D were found to anchor to the Golgi region *via* their cysteine residues or cysteine rich domains^79,80^ and this may also find utility. However, it has been noted that cysteine is too hydrophilic,^81^ and consequently not membrane permeable in some cases. We therefore incorporated an *S*-trityl protected cysteine moiety into a new Zn^2+^ probe through ‘click’ chemistry, and confirmed the probe to be membrane permeable.^82^ Interestingly, the trityl group appeared to be removed *in cellulo* within 24 hours, and the deprotected probe accumulated in the Golgi apparatus.

### Nucleus targeting

The nucleus, as the control centre of the cell, maintains the integrity of genes and controls the activities of the cell by regulating gene expression. In eukaryotic cells, there is a double membrane which encloses the organelle from the cytoplasm. This nuclear membrane is impermeable to large molecules, and the nuclear pores are the channels for large molecules, which must be actively transported by carrier proteins, while also allowing free movement of small molecules and ions. Among these, ZNT9 and ZIP7 were found to be responsible for zinc influx and efflux. About 30–40% of total intracellular zinc is found in the nucleus, which plays an important role in the regulation of cell proliferation. It stabilises the structures of DNA, RNA and the ribosome, and is involved in DNA and protein synthesis. Nuclear hormone receptors are regulated by zinc finger domains, and zinc deficiency impairs their responsiveness, which also has effects on metabolic processes related to growth.^83^ Some zinc-dependent proteins have been found in the nucleus functioning as transporters.^84^ It is therefore necessary to image Zn^2+^ in the nucleus to better understand zinc influx and distribution and its related biological processes.

**Fig. 14:**
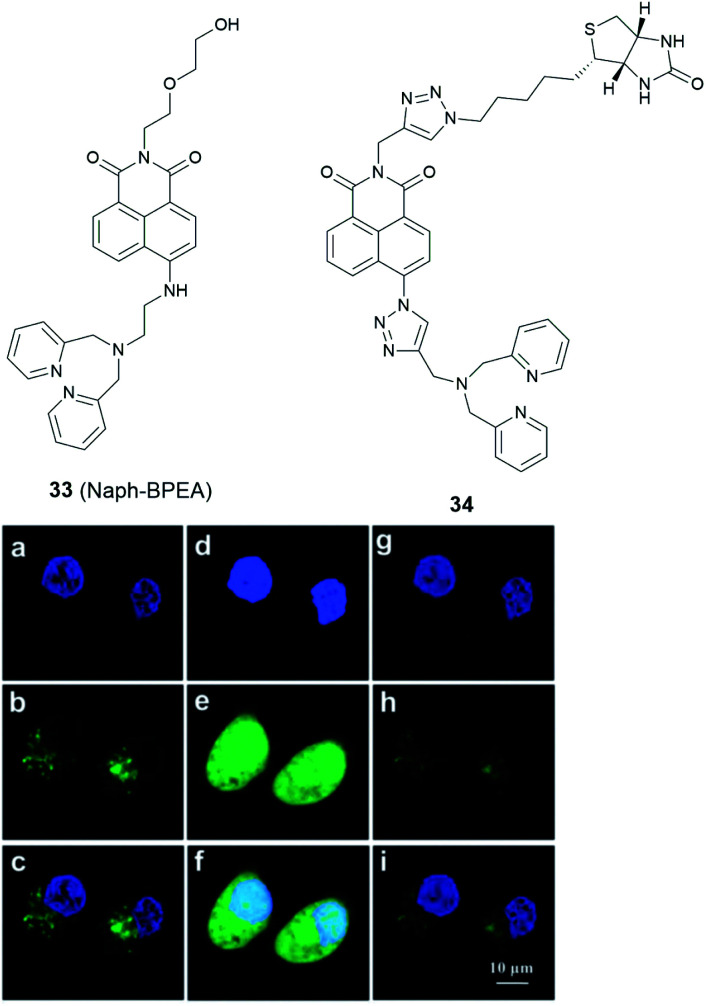
The structure of **33** (Naph-BPEA) and **34**. Confocal fluorescence images of HeLa cells co-stained with Hoechst 33 342 (blue) and **33** (green). (a–c): images of the co-stained cells; (d–f): images of the co-stained cells followed by Zn^2+^-pyrithione incubation; (g–i): images of cells which were subsequently treated by TPEN. Reproduced from ref. 90 with permission from the Royal Society of Chemistry, Copyright [2013].

Nucleus localised Zn^2+^ probes have been realised by genetically encoded sensors^85^ or small molecule fusion protein tags,^86^ however there are no small molecule Zn^2+^ probes showing specific nucleus targeting, though the commercial probes Newport Green^87^ and Zinquin^88^ have been reported to display entire cell distribution, including the nucleus. A fluorescent probe based on naphthalimide, **33** (Naph-BPEA, Fig. 14), reported by Guo *et al.*^89^ displayed a 4-fold fluorescence enhancement and blue shift of the ICT absorption band on Zn^2+^ binding. The intracellular distribution of the probe was studied in HeLa and HepG2 cells and when the cells were stained with **33**, only cytosolic fluorescence was observed. However, when exogenous Zn^2+^ was added, the entire cell became fluorescent, including the nucleus, which was confirmed with the Hoechst stain, a commercial DNA dye (Fig. 14). It demonstrated the ability of the probe to penetrate the nuclear envelope, but the nuclear fluorescence response was small due to the low nuclear concentration of labile Zn^2+^. The authors speculated that incorporation of the 4-amino-1,8-naphthalimide moiety to be the reason for its nuclear envelope penetrability through positive and negative control experiments. Furthermore, *in vivo* Zn^2+^ imaging in zebrafish larva was also performed. In 2018, we developed the biotinylated probe **34** based on 1,8-naphthalimide moiety for imaging Zn^2+^ in breast cancer MCF-7 cells and found it has nuclear envelope penetrability.^90^ However, it suffers from the same issue as **33** in that it was distributed over the entire cell and its sensitivity was insufficient to detect endogenous nuclear Zn^2+^ with the nucleus fluorescence only being switched on after the addition of exogenous Zn^2+^.

Nonetheless, the challenge remains to develop small molecule probes to image Zn^2+^ in the nucleus specifically, where the concentration of mobile Zn^2+^ in this organelle is also very low. In this case, targeting groups that bind to DNA may be possibilities, such as the non-fluorescent pyrrole–imidazole polyamides.^91,92^

## Summary and outlook

Efforts in the development of small molecule fluorescent probes in last two decades have provided a wide range of tools to study Zn^2+^ related processes at the subcellular level, which are already beginning to be utilised to provide answers to questions around the role of this important cation in biology. However, its role, especially its function in individual cellular processes, remains far from being fully understood, and a number of challenges and requirements endure for the further development of small molecule Zn^2+^ probes.

Reliable and effective small molecule probes to study mobile Zn^2+^, as well as a number of other analytes, in some specific organelles, such as the Golgi apparatus and nucleus, are still needed. It should be noted that many factors need to be considered to achieve organelle targeting, including their pH,^93^ ionic strength and temperature of the medium, concentration of the probe and incubation time.^75^ It is also desirable to demonstrate the organelle targeting efficacy of new probes in a wide range of cell lines in case of false positive results in individual cell lines if they are to find widespread utility in biological applications.

There remains a significant demanded to develop tools to accurately quantify the endogenous Zn^2+^ in specific locations by developing ratiometric fluorescent probes, since most of the probes discussed herein are based on the typical PET mechanism and are not able to image Zn^2+^ quantitively due to either no or a small shift in emission wavelength on analyte binding. One viable route towards the development of ratiometric PET probes has been reported by Radford *et al.* in which a second zinc-insensitive fluorophore was appended to a zinc-sensitive PET based probe using a polyproline helix as linker to provide sufficient rigidity.^94^ This approach may open possibilities for other ratiometric organelle targeted probes, although this would further complicate their synthesis and it is possible that such derivatisation may affect cell uptake and targeting properties. As an alternative to PET probes, small molecule ICT probes utilising similar organelle targeting vectors may come to the fore, but again their addition to existing systems adds to synthetic complexity and may adversely affect their photochemical properties.

Probes with longer wavelength excitation and emission profiles, such as in the near-infrared range (650–900 nm), which are finding increasing application *in vivo*, are also required and could be utilised to study Zn^2+^ in deeper tissue with fewer undesirable effects from autofluorescence of surrounding tissues and less harm to biological samples. More importantly, with the rapid development of high spatiotemporal resolution fluorescent microscopic techniques, especially super-resolution microscopy, a number of new challenges are emerging. Besides the basic requirements for biological applications, probes with excellent photostability under the high photon intensity that these techniques utilise are required, whilst also displaying selective organelle targeting ability and a capability to provide real-time information about the location, dynamics and interactions of Zn^2+^ in living cells. In this case, small molecule markers are preferable as they have shown higher photostability compared to fluorescent proteins. The cyanine, BODIPYS, and especially the rhodamines and their derivatives have been successfully applied in super-resolution imaging,^95,96^ which could have potential as fluorophores for organelle specific Zn^2+^ imaging at the nanometre level. In this case, it may also be necessary to develop routinely performed experiments for the assessment of photostability as part of the characterisation of newly reported probes so that they can be compared more easily.

Additionally, the extension of the application of fusion tags, such as SNAP-tag^97^ and HaloTag,^86^ or bioorthogonal reactions could be considered to localise Zn^2+^ probes to an individual protein, to study spatiotemporal zinc fluctuations in a specific process, or its response to intra- or extracellular environmental changes, which may help better understand the mechanism of disease initiation and development due to the dysfunction of zinc homeostasis.

Thus, whilst many exciting developments in small molecule fluorescent probes have occurred in recent years and many significant advances have been made there is still much work to be done and synthetic chemists, collaborating with biologists and biomedical scientists, are likely to be at the vanguard of future developments in the area.

## Conflicts of interest

There are no conflicts to declare.
